# A Novel Online Sequential Extreme Learning Machine for Gas Utilization Ratio Prediction in Blast Furnaces

**DOI:** 10.3390/s17081847

**Published:** 2017-08-10

**Authors:** Yanjiao Li, Sen Zhang, Yixin Yin, Wendong Xiao, Jie Zhang

**Affiliations:** 1 School of Automation & Electrical Engineering, University of Science and Technology Beijing, Beijing 100083, China; liyanjiao2009622@163.com (Y.L.); yyx@ies.ustb.edu.cn (Y.Y.); wdxiao@ustb.edu.cn (W.X.); zhangjie2009622@163.com (J.Z.); 2 Key Laboratory of Knowledge Automation for Industrial Processes, Ministry of Education, Beijing 100083, China

**Keywords:** soft-sensor approach, data-driven model, machine learning, gas utilization ratio, blast furnace, online sequential extreme learning machine

## Abstract

Gas utilization ratio (GUR) is an important indicator used to measure the operating status and energy consumption of blast furnaces (BFs). In this paper, we present a soft-sensor approach, i.e., a novel online sequential extreme learning machine (OS-ELM) named DU-OS-ELM, to establish a data-driven model for GUR prediction. In DU-OS-ELM, firstly, the old collected data are discarded gradually and the newly acquired data are given more attention through a novel dynamic forgetting factor (DFF), depending on the estimation errors to enhance the dynamic tracking ability. Furthermore, we develop an updated selection strategy (USS) to judge whether the model needs to be updated with the newly coming data, so that the proposed approach is more in line with the actual production situation. Then, the convergence analysis of the proposed DU-OS-ELM is presented to ensure the estimation of output weight converge to the true value with the new data arriving. Meanwhile, the proposed DU-OS-ELM is applied to build a soft-sensor model to predict GUR. Experimental results demonstrate that the proposed DU-OS-ELM obtains better generalization performance and higher prediction accuracy compared with a number of existing related approaches using the real production data from a BF and the created GUR prediction model can provide an effective guidance for further optimization operation.

## 1. Introduction

Single-hidden layer feedforward neural networks (SLFNs) have been widely used in many fields because they can approximate any function and form decision boundaries with arbitrary shape if the activation function is properly chosen [1,2,3]. However, traditional machine learning algorithms for training SLFNs such as the back-propagation (BP) algorithm [4,5] can easily get trapped in a local minimum and are time-consuming. To address the aforementioned problems, Huang et al. proposed a new kind of machine learning algorithm named extreme learning machine (ELM) for training SLFNs [6]. The salient features of ELM are that its hidden layer parameters do not require manual intervention and can be assigned randomly before training, and the output weight is determined analytically via the least squares estimation method, making it easy to implement with better generalization performance and faster learning speed [7,8,9]. Nowadays, ELM has been widely used in many fields, such as landmark recognition [10], industrial production [11], electronic nose [12], localization [13,14], etc.

ELM was initially proposed for batch learning, which considers all the available data at once. However, in a great number of real world applications, data may be received sequentially. In such cases, sequential learning should be preferable to batch learning. For this reason, the online learning type of ELM was proposed by Liang et al. and named online sequential ELM (OS-ELM) [15]. OS-ELM can deal with data of different sizes arriving one-by-one or chunk-by-chunk to update the training model in each time step. Recently, many improved learning algorithms based on OS-ELM were proposed by Lan et al. [16], Deng et al. [17], Huang et al. [18], Matias et al. [19]. However, these variants do not consider the data saturation problem, which is caused by some data generated in time-varying environments, thus there are also many improved OS-ELM-based algorithms for dealing with time-varying data. In [20], OS-ELM with kernels (OS-ELMK) was proposed by Wang et al., in which a limited memory strategy was designed to remove the data that have been trained when the number of trained data exceeds a predetermined threshold. Zhao et al. [21] proposed an OS-ELM with forgetting mechanism (FOS-ELM) to learn the data generated in time-varying environments, they implemented fixed sliding window to discard the old data when the new ones arrived. With the arrival of new observations, the sliding window and limited memory strategy ignore the contribution of the old data to the model, thus they cannot be adapted well to time-varying environments. Considering the problem from another point of view, the concepts of forgetting factor and weight strategy were introduced. Gu et al. [22] developed timeliness OS-ELM (TOS-ELM), which brought an adaptive weight strategy and iteration scheme to OS-ELM so that the newly arriving data could be given a reasonable attention to represent the current situation. However, TOS-ELM is time-consuming due to its iteration scheme. Lim et al. [23] presented an adaptive forgetting factor for OS-ELM and illustrated a complex mechanism to calculate the forgetting factor based on a gradient descent method, but it has a relatively high additional computational complexity and is also time-consuming. In addition, all these listed methods update the training model in each time step during the learning process, which would increase the computational complexity. Actually, when the system is relatively stable or the current training model can meet the prediction requirement, it may not need to update in each time step. In view of this fact, an appropriate model updated strategy should be considered to reduce the computational complexity and time consumption.

In order to tackle these issues, we propose a novel OS-ELM referred to as DU-OS-ELM. In DU-OS-ELM, a novel dynamic forgetting factor (DFF) and updated selection strategy (USS) are proposed to tackle the time-varying sequential data. The motivation for the DFF is that larger estimation errors should employ smaller forgetting factor to adapt to the time-varying environments and smaller estimation errors should employ larger forgetting factor to increase memory length at stable situations. In this way, the old collected data are forgotten gradually and the newly acquired data are given more considerable attention. Taking advantage of the novel DFF can effectively accelerate the convergence speed of DU-OS-ELM with low computational complexity. In addition, we also develop an USS to judge whether the model should be updated with the newly coming data in each time step. Thus, the proposed approach is more in line with the actual production situation. In addition, the convergence analysis of the proposed DU-OS-ELM is presented to ensure the estimation of the output weight converge to the true value with the new data arriving.

The blast furnace (BF) ironmaking process is the primary link in iron and steel production and one of its main energy-consuming processes [24,25,26]. Due to its high energy consumption, energy-saving and consumption-reduction are currently becoming important issues in BF ironmaking [27]. In actual production, the gas utilization ratio (GUR) is an important indicator for determining the energy consumption and operating status [28]. The improvement of GUR is an important embodiment of technical progress, therefore, the reasonable prediction and control of GUR is significant for efficient and low consumption BF iron production. However, due to the high complexity of the furnace interior, GUR is difficult to understand and evaluate through only the rich experience of specialized operators, therefore, how to establish an accurate GUR prediction model has become a hot research topic. Recently, several researchers are engaged in addressing this issue, mainly including mechanism-based models and data-driven models. For mechanism-based models, taking into account the influence of BF on GUR, a comprehensive improvement strategy based on production mechanism was proposed to enhance the GUR [29]. Due to the fast development of measurement instruments and sensor techniques, one can obtain a large amount of data from the BF ironmaking process. Therefore, data-driven models using machine learning approaches are an efficient way for predicting GUR. Based on expert theory and practical experience, a linear regression model between operating parameters and GUR was established [30]. Xiao et al. proposed a GUR forecasting model based on an improved BP algorithm [31]. An improved support vector regression (SVR) model based on the correlation analysis of some influence factors and GUR [32], and a chaos radial basis function (RBF) model [33] for GUR prediction were proposed, respectively. Xiao et al. presented a chaotic prediction method to analyze the characteristics of GUR [34]. Besides, many methods have been employed to establish soft-sensor models to predict the hot metal silicon content in the BF. A pruning algorithm is implemented to find both relevant inputs with their corresponding time delay and appropriate network connectivity based on a feedforward neural network to improve the prediction accuracy of hot metal silicon content in [35]. Support vector machine (SVM) and least square support vector machine (LSSVM) are not only applied to the value prediction of hot metal silicon content, but also for the prediction of change trends [36,37,38]. Although the aforementioned studies have obtained relatively satisfactory achievement, most of these models are constructed based on mechanistic research and fixed learning samples, which ignore the fact that BF is a time-varying system, and they are not adequate for online use. To tackle this issue, the proposed DU-OS-ELM is employed to establish an online GUR prediction model.

In this paper, a modified OS-ELM algorithm named DU-OS-ELM is firstly proposed based on DFF and USS. Compared with existing approaches, the proposed DU-OS-ELM is more suitable for dealing with data generated in time-varying environments and can meet the real-time requirements of real world applications. Then a soft-sensor model is established based on the proposed DU-OS-ELM to implement a fast and accurate GUR prediction. The experimental results indicate that DU-OS-ELM can achieve better performance than some similar algorithms using real industrial data. This research can help BF operators understand the energy consumption well, according to the changes of GUR, which provides effective guidance for further optimization operation.

The remaining parts of this paper are arranged as follows: Section 2 gives some preliminaries, including a brief introduction of ELM and OS-ELM. The proposed DU-OS-ELM is detailed in Section 3, including the DFF mechanism, USS and the convergence analysis of the proposed approach. The soft-sensor model for GUR is presented in Section 4. Section 5 presents the performance evaluation of DU-OS-ELM using the real data collected from a BF. Discussions and conclusions are given in Section 6 and Section 7, respectively.

## 2. Preliminaries

In this section, ELM and OS-ELM are introduced to facilitate the understanding of the proposed DU-OS-ELM.

### 2.1. Extreme Learning Machine

**Figure 1:**
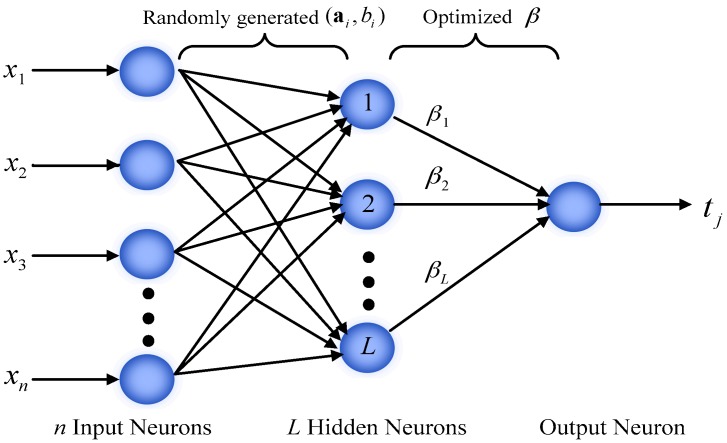
Basic architecture of ELM.

As shown in Figure 1, ELM has a three-layer architecture. Considering *N* training samples $(x_{i},t_{i})\in R^{n}\times R^{m}$, here $x_{i}={\left[x_{i1},x_{i2},\ldots ,x_{in}\right]}^{T}$ represents a *n*-dimensional attributes of the *i*th sample and $t_{i}={\left[t_{i1},t_{i2},\ldots ,t_{im}\right]}^{T}$ is a *m*-dimensional target vector [12]. The output of the network with *L* hidden nodes can be calculated by:

(1) $$\sum_{i=1}^{L}\beta_{i}G(a_{i},b_{i},x_{i})=t_{j},j=1,\ldots ,N$$

where $a_{i}=[a_{i1},a_{i2},\cdots ,a_{in}]$ and $b_{i}$ denote the learning parameters of the *i*th hidden node. $\beta_{i}=[\beta_{i1},\beta_{i2},\ldots ,\beta_{im}]$ is weight vector connecting the *i*th hidden node and the output nodes, G(●) is the *i*th hidden node output function.

Equation (1) can be further compactly written in matrix form as:

(2) Hβ=T

where H is the hidden layer output matrix:

(3) $$H(a_{1},\ldots ,a_{L},b_{1},\ldots b_{L},x_{1},\ldots ,x_{N})={\left[\begin{array}{ccc} G(a_{1},b_{1},x_{1}) & \cdots & G(a_{L},b_{L},x_{1})\\ \vdots & \ddots & \vdots \\ G(a_{1},b_{1},x_{N}) & \cdots & G(a_{L},b_{L},x_{N}) \end{array}\right]}_{N\times L}$$

(4) $$\beta ={\left[\begin{array}{c} \beta_{1}^{T}\\ \vdots \\ \beta_{L}^{T} \end{array}\right]}_{L\times m}\;\mathrm{and}\;T={\left[\begin{array}{c} t_{1}^{T}\\ \vdots \\ t_{N}^{T} \end{array}\right]}_{N\times m}$$

The following optimization problem is formulated to identify β:

(5) $$\begin{array}{l} \min:J_{ELM}=\frac{1}{2}{\left\|\beta \right\|}^{2}+\frac{\gamma }{2}\sum_{i=1}^{N}{\left\|e_{i}\right\|}^{2}\\ s.t.:h(x_{i})\beta =t_{i}^{T}-e_{i}^{T} \end{array}$$

where γ is the regularization parameter, $e_{i}$ is the training error.

We can convert the above optimization problem into a dual optimization problem by employing the Karush-Kuhn-Tucker (KKT) theorem and further the output weight β can be obtained as:

(6) $$\beta ={\left(\frac{I}{\gamma }+H^{T}H\right)}^{-1}H^{T}T$$

(7) $$\mathrm{or}\;\beta =H^{T}(\frac{I}{\gamma }+HH^{T})T$$

For computational convenience, when N>L, the solution of Equation (6) is preferred, and when N<L, we prefer to use the solution of Equation (7) to estimate β.

### 2.2. Online Sequential Extreme Learning Machine

The online version of ELM, which does not need to retrain the model with all the observations, but instead updates the model only with the newly arriving data was proposed by Liang et al. and named OS-ELM [15]. OS-ELM is similar to recursive least squares (RLS) based on the matrix inversion lemma to estimate the output weight vector [39,40]. OS-ELM consists of two phases: the initialization phase (a small-scale ELM) and the sequential learning phase.

For the initial data ${ℵ}_{0}=\{x_{j},t_{j}|j=1,\ldots ,N_{0}\}$, $N_{0}\geq L$ in the initialization phase. According to the ELM theory, the output weight is initialized by:

(8) $$\beta^{\left(0\right)}=P_{0}H_{0}^{T}T_{0}$$

where $T_{0}=[t_{1},t_{2},\cdots ,t_{N_{0}}]$ and $P_{0}={\left(\frac{I}{\gamma }+H_{0}^{T}H_{0}\right)}^{-1}$.

In the sequential learning phase, the output weight is computed using an iteration technique because more and more observations arrive continuously. When the (*k* + 1)th chunk of the new observation: ${ℵ}_{k+1}=\{x_{j},t_{j}|j=(\sum_{j=0}^{k}N_{j})+1,\ldots ,\sum_{j=0}^{k+1}N_{j}\}$ is presented, where $N_{k+1}$ denotes the number of newly arriving data in the (*k* + 1)th chunk, the new matrices $H_{k+1}$ and $\beta^{\left(k+1\right)}$ in the (*k* + 1)th are calculated as follows:

(9) $$K_{k+1}=\frac{P_{k}H_{k+1}^{T}}{I+H_{k+1}P_{k}H_{k+1}^{T}}$$

(10) $$P_{k+1}=P_{k}-K_{k+1}H_{k+1}P_{k}$$

(11) $$e_{k+1}=T_{k+1}-H_{k+1}\beta^{\left(k\right)}$$

(12) $$\beta^{\left(k+1\right)}=\beta^{\left(k\right)}+P_{k+1}H_{k+1}^{T}e_{k+1}$$

For further details of this updating method readers may refer to [3,15]. Thus, the output estimation function for OS-ELM is given by:

(13) $$f_{OS-ELM}(x)=\sum_{i=1}^{L}\beta_{i}^{\left(k+1\right)}G(a_{i},b_{i},x_{i})$$

## 3. Details of the Proposed DU-OS-ELM

In real world applications, the production process is continuous and unstable with time, that is to say, the acquired data are changing. In order to better adapt to this situation, the contribution of the newly arriving data should be greater than that of previous ones. In addition, with learning ongoing and new incremental data for OS-ELM arriving sequentially, the storage space for the matrix will increase infinitely, which will lead to a loss of weight correction ability so that a data saturation problem finally appears. Therefore, we put forward the novel DFF and USS for OS-ELM to enhance the performance of sequential learning in time-varying environments.

### 3.1. Dynamic Forgetting Factor

The original optimization problem of OS-ELM can be represented by Equation (5). The purpose of adding the forgetting factor is to minimize the norm of the output weight and the weighted sum of squared error, so Equation (5) becomes:

(14) $$\begin{array}{l} \min:L=\frac{1}{2}{\left\|\beta \right\|}^{2}+\frac{\gamma }{2}\sum_{i=1}^{t}\lambda^{t-i}e_{i}^{2}\\ s.t.:h(x_{i})\beta =t_{i}^{T}-e_{i}^{T} \end{array}$$

Thus, we can obtain the similar expression with RLS:

(15) $$K_{k+1}=\frac{P_{k}H_{k+1}^{T}}{\lambda +H_{k+1}P_{k}H_{k+1}^{T}},$$

(16) $$P_{k+1}=\frac{1}{\lambda }(P_{k}-K_{k+1}H_{k+1}P_{k}),$$

(17) $$e_{k+1}=T_{k+1}-H_{k+1}\beta^{\left(k\right)},$$

(18) $$\beta^{\left(k+1\right)}=\beta^{\left(k\right)}+P_{k+1}H_{k+1}^{T}e_{k+1},$$

where λ∈(0,1] is the forgetting factor.

In the practical engineering problems, the dynamic characteristics of the system are not always changing with the same rules. For these kinds of situations, a constant forgetting factor will not ensure the satisfactory performance of the model. Therefore, we should automatically adjust the forgetting factor with the dynamic changes of the characteristic to obtain more accurate results. When the system parameters mutate, we select a smaller forgetting factor, which makes the model track the changing trend of the characteristics quickly. On the contrary, by choosing a larger forgetting factor to increase the memory length in the stable status and improve the accuracy of the model. Therefore, we present a novel DFF to improve the dynamic tracking ability of the original OS-ELM when it needs to update.

The essence of the novel DFF is to adjust λ to minimize the mean square error (MSE) and increase the convergence speed. Hence, we develop the DFF based on the predicted errors, which are updated recursively as:

(19) $$\lambda_{k}=\lambda_{\min}+(1-\lambda_{\min})\times e^{-\mu \left\|e(k)\right\|},$$

where $\lambda_{k}$ is the forgetting factor for the *k*th chunk of new observations, μ is the step size to control the rate of λ to trend to 1, which will be discussed later. The additional computational complexity with respect to the original OS-ELM is only a few arithmetic calculations.

We set the boundary constraint of the DFF to avoid it too large or too small. The constraint conditions are as follows [41]:

(20) $$\lambda_{k}=\{\begin{array}{c} \lambda_{\min},\;if\;\lambda_{k}<\lambda_{\min}\\ \lambda_{\max},\;if\;\lambda_{k}>\lambda_{\max} \end{array}$$

**Figure 2:**
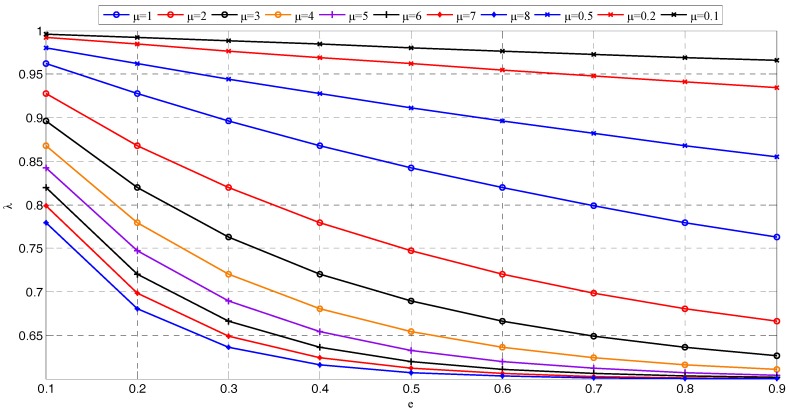
Changes of λ with different μ.

Trivially, the forgetting factor in the exponential window can be used to control the effective memory of the window. λ can be written as $\lambda =e^{-1/\tau }$, where τ is the effective time constant that is related to data memory length. Thus, $\lambda_{\max}$ is set as 0.999 when τ=1000, and $\lambda_{\min}$ is set as 0.6 when τ=2 [42]. Figure 2 presents the changes of λ in the case of different μ. We can find that λ has a faster rate approaching 1 with the smaller μ. Under the same error, the larger μ usually has a corresponding smaller λ, thus, the memory length becomes shorter. In addition, with the decrease of μ, the change of λ is more stable. Therefore, we should determine μ according to the different data characteristics.

The behavior of the DFF is as follows: the distribution of the data in the time-varying environment influences the calculation of $\lambda_{k}$. When the system parameters are fluctuating, e(k) is increased, and $\lambda_{k}$ is decreased according to Equation (19). On the other hand, the impact of the old observations is reduced in the calculation of the output weight $\beta^{\left(k+1\right)}$ in Equation (18).

### 3.2. Updated Selection Strategy

In brief, when the new data arrive one-by-one or chunk-by-chunk for the OS-ELM training model, the output weight needs to be updated. However, when the production process is stable or the current model can meet the prediction requirements, there is no need to update the existing model in every time step [43]. Thus, a criterion is added to judge whether the training model needs to be updated:

(21) $$\beta^{\left(k+1\right)}=\{\begin{array}{c} \beta^{k}+P_{k+1}H_{k+1}^{T}e_{k+1},e_{k+1}>e_{k}\\ \beta^{k},e_{k+1}\leq e_{k} \end{array}.$$

If the predicted error of the new data by the current model is larger than the previous one, the existing model needs to be updated. Otherwise, the existing model does not need to be updated. Thus, this can not only reduce the time consumption, but also avoid the accumulation of errors.

### 3.3. Convergence Analysis

We present the convergence analysis of the proposed approach in this subsection.

**Theorem** **1.** *Consider the proposed DU-OS-ELM and its output weight updated process, the estimation of output weight* ${\hat{\beta }}^{\left(k\right)}$ *will converge to the true value* $\beta^{\left(k\right)}$. *In addition, the output weight updated error has following upper bounds:*

(22) $$E\left[{\left\|{\tilde{\beta }}^{\left(k\right)}\right\|}^{2}\right]\leq \lambda_{\max}^{2}{\left(C/\prod_{i=1}^{k-1}\lambda_{i}\right)}^{2}\sigma_{0}\delta_{0},$$

*where* ${\tilde{\beta }}^{\left(k\right)}={\hat{\beta }}^{\left(k\right)}-\beta^{\left(k\right)}$, $\sigma_{0}=\xi_{\max}^{2}(P_{0}^{-1})$, $C=\xi_{\max}(P_{0})$, *and* $\delta_{0}=E\left[{\left\|{\tilde{\beta }}^{\left(0\right)}\right\|}^{2}\right]$. $\xi_{\max}(x)$ *is the maximum eigenvalue of the matrix* x.

**Proof.**  Define the output weight updated error vector as:

(23) $${\tilde{\beta }}^{\left(k\right)}={\hat{\beta }}^{\left(k\right)}-\beta^{\left(k\right)}.$$

Using Equations (2), (15), (17) and (18), we can obtain:

(24) $${\tilde{\beta }}^{\left(k\right)}=(I-\frac{P_{k-1}H_{k}^{T}H_{k}}{\lambda_{k}+H_{k}P_{k-1}H_{k}^{T}}){\tilde{\beta }}^{\left(k-1\right)}.$$

According to Equations (16) and (24), we have:

(25) $$P_{k}^{-1}{\tilde{\beta }}^{\left(k\right)}=\lambda_{k}P_{k-1}^{-1}{\tilde{\beta }}^{\left(k-1\right)}.$$

Thus:

(26) $${\tilde{\beta }}^{\left(k\right)}=\lambda_{k}P_{k}^{-1}P_{k-1}^{-1}{\tilde{\beta }}^{\left(k-1\right)}=\prod_{i=1}^{k}\lambda_{i}P_{k}P_{0}^{-1}{\tilde{\beta }}^{\left(0\right)}.$$

Consider the update of $P_{k}^{-1}$, then:

(27) $$P_{k}^{-1}=\lambda_{k}P_{k-1}^{-1}+H_{k}^{T}H_{k}.$$

From which we have:

(28) $$\xi_{\min}(P_{k}^{-1})\geq \lambda_{k}\xi_{\min}(P_{k-1}^{-1})\geq \cdots \geq \prod_{i=1}^{k}\lambda_{i}\xi_{\min}(P_{0}^{-1}).$$

Assuming $\xi_{\max}(P_{0})\leq C$, the Equation (28) becomes:

(29) $$\xi_{\max}(P_{k-1})\leq \frac{1}{\prod_{i=1}^{k-1}\lambda_{i}}\xi_{\max}(P_{0})\leq \frac{C}{\prod_{i=1}^{k-1}\lambda_{i}}.$$

Furthermore, for $0<\lambda_{k}<1$, the DFF satisfies:

(30) $$0<{\left(\lambda_{\min}\right)}^{k}\leq \prod_{i=1}^{k}\lambda_{i}\leq {\left(\lambda_{\max}\right)}^{k}\leq \lambda_{\max}<\infty ,$$

then:

(31) $$\begin{array}{cc} E\left[{\left\|{\tilde{\beta }}^{\left(k\right)}\right\|}^{2}\right] & =E\left[{\left\|\prod_{i=1}^{k}\lambda_{i}P_{k}P_{0}^{-1}{\tilde{\beta }}^{\left(0\right)}\right\|}^{2}\right]\\ & ={\left(\prod_{i=1}^{k}\lambda_{i}\right)}^{2}E\left[{\left\|P_{k}P_{0}^{-1}{\tilde{\beta }}^{\left(0\right)}\right\|}^{2}\right]\\ & \leq {\left(\prod_{i=1}^{k}\lambda_{i}\right)}^{2}\xi_{\max}^{2}(P_{k})\xi_{\max}^{2}(P_{0}^{-1})E\left[{\left\|{\tilde{\beta }}^{\left(0\right)}\right\|}^{2}\right]\\ & \leq \lambda_{\max}^{2}{\left(C/\prod_{i=1}^{k-1}\lambda_{i}\right)}^{2}\sigma_{0}\delta_{0} \end{array}.$$

This completes the proof of this theorem. ☐

In addition, we discuss the error limitation. Define a non-negative function $S_{k}$ as:

(32) $$S_{k}=\frac{{\left[{\tilde{\beta }}^{\left(k\right)}\right]}^{T}P_{k}^{-1}{\tilde{\beta }}^{\left(k\right)}}{\prod_{i=1}^{k}\lambda_{i}}.$$

Then, we have:

(33) $$\begin{array}{cc} S_{k}-S_{k-1} & =\frac{{\left[{\tilde{\beta }}^{\left(k\right)}\right]}^{T}P_{k}^{-1}{\tilde{\beta }}^{\left(k\right)}}{\prod_{i=1}^{k}\lambda_{i}}-\frac{{\left[{\tilde{\beta }}^{\left(k-1\right)}\right]}^{T}P_{k-1}^{-1}{\tilde{\beta }}^{\left(k-1\right)}}{\prod_{i=1}^{k-1}\lambda_{i}}=\frac{1}{\prod_{i=1}^{k-1}\lambda_{i}}{\left({\tilde{\beta }}^{\left(k\right)}-{\tilde{\beta }}^{\left(k-1\right)}\right)}^{T}P_{k-1}^{-1}{\tilde{\beta }}^{\left(k-1\right)}\\ & =-\frac{1}{\prod_{i=1}^{k-1}\lambda_{i}}\left(\frac{{\left[{\tilde{\beta }}^{\left(k-1\right)}\right]}^{T}H_{k}^{T}H_{k}{\tilde{\beta }}^{\left(k-1\right)}}{\lambda_{k}+H_{k}P_{k-1}H_{k}^{T}}\right)=-\frac{e_{k}^{2}}{\prod_{i=1}^{k}\lambda_{i}+\prod_{i=1}^{k-1}\lambda_{i}H_{k}P_{k-1}H_{k}^{T}}\\ & \leq -\frac{e_{k}^{2}}{\prod_{i=1}^{k}\lambda_{i}+\prod_{i=1}^{k-1}\lambda_{i}\xi_{\max}(P_{k-1}){\left\|H_{k}\right\|}^{2}}\leq 0 \end{array}.$$

From Equation (33), i.e., $S_{k}-S_{k-1}\leq 0$, it is easy to see that $S_{k}$ converges, then we can obtain:

(34) $$\underset{k\to \infty }{\lim}\frac{e_{k}^{2}}{\prod_{i=1}^{k}\lambda_{i}+\prod_{i=1}^{k-1}\lambda_{i}\xi_{\max}(P_{k-1}){\left\|H_{k}\right\|}^{2}}=0.$$

Substituting Equation (29) into (34), we have:

(35) $$\underset{k\to \infty }{\lim}\frac{e_{k}^{2}}{\prod_{i=1}^{k}\lambda_{i}+C{\left\|H_{k}\right\|}^{2}}=0,$$

where $\prod_{i=1}^{k}\lambda_{i}>0$, ${\left\|H_{k}\right\|}^{2}>0$, so we obtain $\underset{k\to \infty }{\lim}e_{k}=0$.

As mentioned above, we can summarize the proposed DU-OS-ELM in the following steps:

Step 1: Determine the model parameters by initial data $S_{0}=\{x_{j},t_{j}|j=1,\ldots ,N_{0}\}$, such as hidden nodes number L, activation function $G(a_{i},b_{i},x_{i})$ and the initial value of the DFF;
Step 2: Randomly generate the hidden layer parameters ($a_{i}$, $b_{i}$);
Step 3: Calculate the initial hidden layer output matrix $H_{0}$ and the initial output weight $\beta^{\left(0\right)}$ by Equation (8), then generate the initial model;
Step 4: Calculate the predicted error of the newly incremental data by the current model;
Step 5: Judge whether the model should be updated by Equation (21), if the model needs not to be updated, the new data will be added directly and the output weight will remain unchanged. If the model needs to be updated, $\lambda_{k}$ is calculated by Equation (19) and then the output weight will be recalculated by Equation (18);
Step 6: Output the final output weight $\beta^{\left(k+1\right)}$.

It should note that the steps 4–5 are used for sequential learning, so the algorithm will repeat these two steps when there are newly arriving data.

## 4. Soft-Sensor Model for GUR Prediction in BF Based on DU-OS-ELM

**Figure 3:**
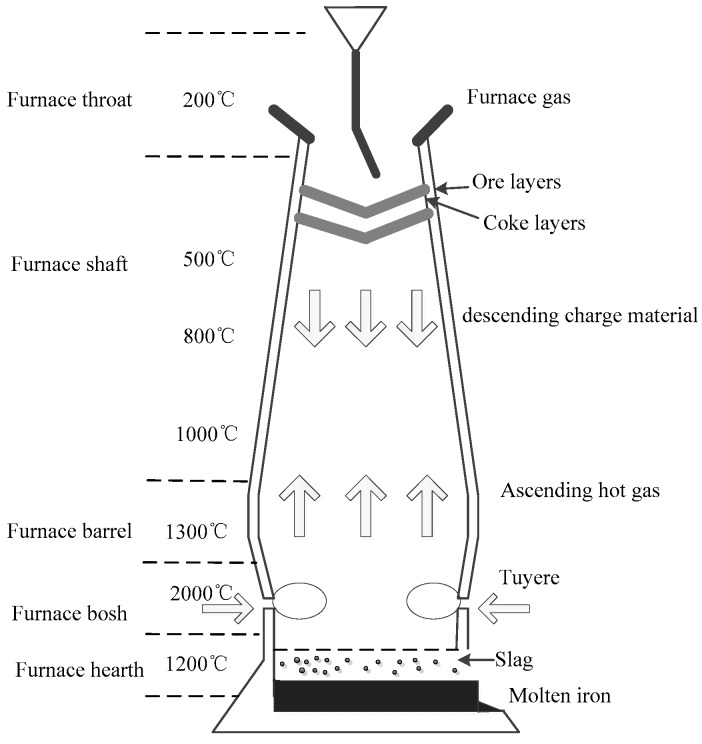
Blast furnace ironmaking process.

As shown in Figure 3, a BF is essentially a large countercurrent heat exchanger and chemical reactor. The reactants including iron ore, coke and limestone are fed into the top layer by layer. In addtion, preheated air and additional oxygen are occasionally blown into the bottom through the tuyeres. A series of complicated chemical reactions and heat transport phenomena occur in the different zones throughout the BF under the different temperatures due to the downward movement of raw materials and the upward flow of hot gases, which can produce a large amount of heat energy that can make the temperature of a BF reach about 2000 °C. The molten pig iron and slag are produced as separating output streams at certain intervals as the production continues. The whole ironmaking cycle will take about 6–8 h [37,44]. The main chemical reactions during the ironmaking process are described by the following equations [25]:

(36) $$3Fe_{2}O_{3}+CO\overset{450{}^{\circ}C}{\to }2Fe_{3}O_{4}+CO_{2},$$

(37) $$Fe_{3}O_{4}+CO\overset{600{}^{\circ}C}{\to }3FeO+CO_{2},$$

(38) $$FeO+CO\overset{\geq 700{}^{\circ}C}{\to }Fe+CO_{2},$$

(39) $$FeO+C\overset{\geq 700{}^{\circ}C}{\to }Fe+CO.$$

Meanwhile, the coke descends downward and reacts with the upwardly flowing preheated air to produce heat immediately:

(40) $$C+O_{2}\to CO_{2}.$$

Then, the carbon dioxide reacts with excess carbon to produce carbon monoxide with high temperature:

(41) $$CO_{2}+C\to 2CO.$$

According to the above analysis, carbon monoxide plays a major role when the iron ore is reduced. Thus, the degree of utilization of carbon monoxide directly affects the progress of the chemical reactions occurring inside the furnace and affects the energy consumption of each ton of iron. Therefore, GUR is an important indicator of energy consumption and operating status in BF iron-making processes. The existing studies on GUR prediction mainly focus on mechanism analysis and fixed leaning samples modeling. However, the BF ironmaking process is multi-variable, nonlinear, time-variant and high complex in nature [45], therefore, it is difficult to implement fast and accurate GUR predictions with the existing models and they are not adequate for online use. Besides, due to the fast development of measurement instruments and sensor techniques, we can obtain a large number of data from BF ironmaking processes. Therefore, we propose an online soft-sensor model for GUR prediction based on the proposed DU-OS-ELM.

There are many parameters that can influence GUR due to BFs’ high complexity. It should be noted that the selection of too many parameters as input variables may increase the complexity of model while too few parameters will degrade the model performance. Hence, the influence of parameter selection for a GUR prediction model plays an important role in improving the model accuracy. The GUR depends on the chemical reactions inside the furnace during the ironmaking process. Blast temperature is the power of the BF gas upward movement. The higher the blast temperature is, the greater the heat blowing into the BF. Thus, increasing the blast temperature can effectively promote the chemical reactions and provide temperature compensation. Under the impact of the pressure difference between the blast pressure and top pressure, gas flows from the bottom upwards to ensure that the chemical reactions between the ore and the coke are carried out normally. If the top pressure increases, the gas velocity will slow down and chemical reactions occur more fully. Blast pressure and blast volume are usually characterized by the permeability index, which has a significant influence on the block zone and gas flow distribution. The top temperature directly reflects the degree of heat exchange between the gas flow and the furnace charge. The purpose of oxygen enrichment is to increase the oxygen content in the air, which contributes to the chemical reaction processes. Blast velocity affects the activity degree of the hearth, the size of the combustion zone and gas flow distribution. Injection of fuels can provide more reducing agents and heat, and also promote the progress of indirect reduction reactions. Thermal load can reflect the reactions between the edge charge and gas. Based on BF expert knowledge and the mechanism of the iron production process, 10 parameters, i.e., blast temperature, blast pressure, blast volume, top pressure, top temperature, permeability index, oxygen enrichment percentage, blast velocity, injecting coal rate, and thermal load, are chosen as the candidate inputs features for modeling. Simultaneously, a grey relational analysis (GRA) method [46] is used to analyze the correlation between these parameters and GUR. The required input variables and correlation analysis results by GRA are listed in Table 1.

**Figure 4:**
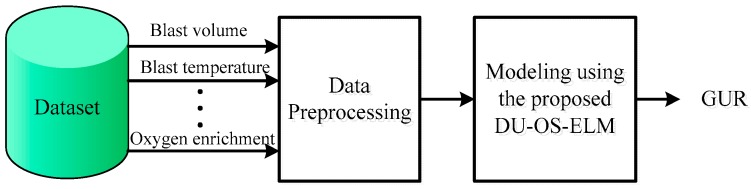
Main frame of the soft-sensor model for GUR prediction.

The greater the grey correlation grades between these parameters and GUR, the stronger the correlation degree is. Here we choose the input parameters with relatively high correlation with GUR. According to the experiential knowledge and results obtained by GRA, the parameters with correlation grades greater than 0.7, i.e., blast temperature, blast pressure, blast volume, top pressure, top temperature, permeability index, and oxygen enrichment percentage, are selected as the final input variables of proposed soft-sensor model, and GUR is the only output variable. Eventually, the proposed DU-OS-ELM is applied to establish the GUR prediction model, which is illustrated in Figure 4.

## 5. Simulation Results and Analysis

In this section, we present the simulation results of the proposed DU-OS-ELM applied to the GUR prediction model. In addition, we compare the performance of DU-OS-ELM with ELM, OS-ELM and FOS-ELM. All the data in the experiments are from the actual production of a steel works located in China. Besides, in order to verify the validity of the USS, we employ a DU-OS-ELM without the USS as the comparison, which is named DOS-ELM. The sigmoidal additive activation function, i.e., G(a,b,x)=1/(1+exp(−(a⋅x+b))), is adopted in the following experiments, where the input weights and the bias are randomly chosen from the range [−1, 1]. All the experiments are carried out in MATLAB 7.11.0 environment running on a desktop computer equipped with an AMD Athlon(tm)IIX2 250 processor operating at 3.00 GHz and with 2.00 GB RAM.

**Remark** **1.** In order to further illustrate the effectiveness of proposed DU-OS-ELM, we apply DU-OS-ELM to the well-known time series prediction problem and hot metal temperature prediction problem. The detailed description of these simulation results which is omitted here for sake of brevity is presented in Appendix A and Appendix B.

### 5.1. Data Preprocessing

The data are collected from a medium-size BF with an inner volume of about 2500 m^3^. We collect 1500 data pairs in all after processing the outlier values. The first 1200 input-output instances are used for training while the others are used for testing to evaluate the performance of the model.

**Figure 5:**
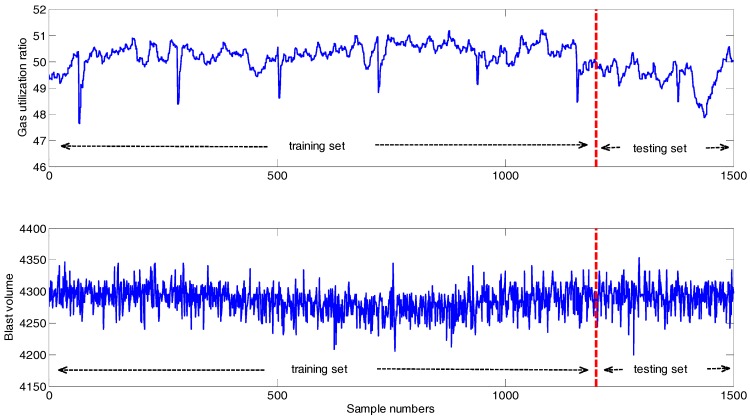
Series of GUR and blast volume in BF.

Next, we calculate some important statistical properties of the variables on the selected data and make the further analysis to get deeper understanding. The statistical properties include maximum, minimum, mean, standard deviation (SD). The results are shown in Table 2 and Table 3, respectively. Table 2 illustrates the statistical properties of GUR in respect of the divided training and testing sets. According to Table 2, the training and testing sets have different statistical properties, so it can better explain the performance of the predicted results. Table 3 details the statistical properties of the variables. Based on the statistical properties, it can be found that the selected data has the characteristics of violent fluctuation. Figure 5 demonstrates the series of GUR and blast volume measured from BF. According to Table 3 and Figure 5, the magnitudes of the variables have big difference clearly. In fact, the effect of the variables with a large magnitude on the modeling is larger than the one with a small magnitude, thus it is not appropriate to directly take the data to establish the model [47]. Thus, all the data are normalized into (0, 1) with the same magnitude to eliminate the influence of dimension among variables before applying in the experiments. The method can be referred Equation (A3) in the Appendix C.

### 5.2. Performance Evaluation

**Figure 6:**
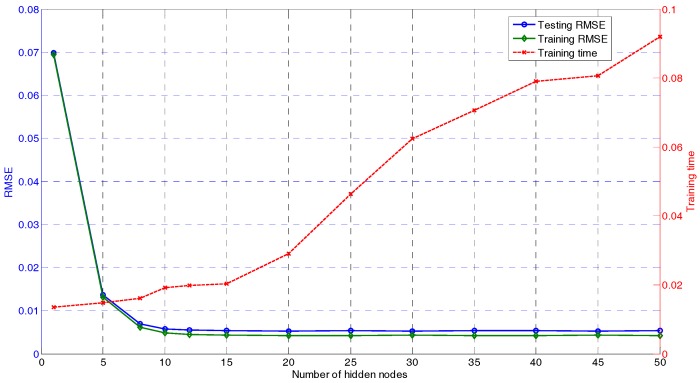
Selection of the hidden nodes number.

To construct the GUR prediction model using the proposed DU-OS-ELM, it is necessary to determine the related parameters. In DU-OS-ELM, there are three important parameters which need to be determined, i.e., the regularization factor γ, the number of hidden nodes *L*, and the step size μ for controlling the forgetting rate. After some experiments we set γ=10 in this paper. The number of hidden nodes with the minimum training error is taken as the optimal *L* [14]. The model selection of the optimal *L* for DU-OS-ELM is shown in Figure 6. According to Figure 6, the green and blue lines on the left stand for the training and testing errors (averaged over 50 trials), and the red line on the right stands for the training time, respectively. In this simulation, we increase *L* from 1 to 50. Accordingly, the intervals from 1 to 15 are relatively small and the hidden nodes increase 5 by 5 from 15 to 50. As shown in Figure 6, with the increase of *L*, the root mean squared error (RMSE) of the model is smaller gradually and the training time is increased. The lowest testing error is achieved when *L* is within the range (15, 50) with smooth RMSE curves. Therefore, one can select the optimal *L* from this range. Considering the computational complexity and testing accuracy, we choose *L* equals to 20 as a good trade-off.

**Figure 7:**
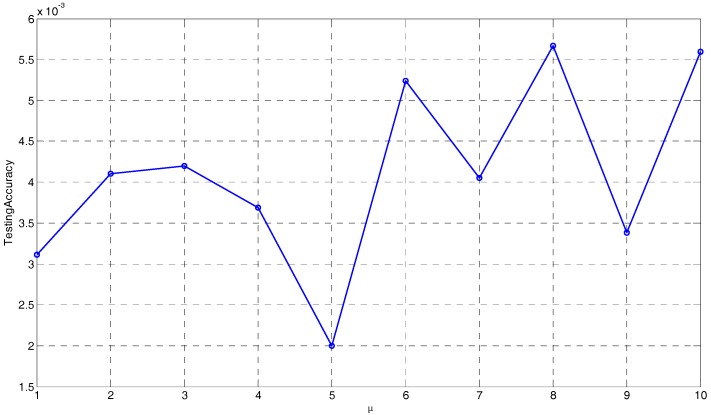
Selection of step size μ.

One of the contributions of the proposed DU-OS-ELM is the utilization of a novel DFF. In Equation (19), the step size μ is an important user specified parameter that also needs to be determined. Figure 7 presents the testing accuracy with different step sizes μ in the range [1, 10]. We increase μ 1 by 1. As observed from Figure 7, the minimum testing accuracy is obtained when μ equals 5, which will be used in the following experiments.

**Figure 8:**
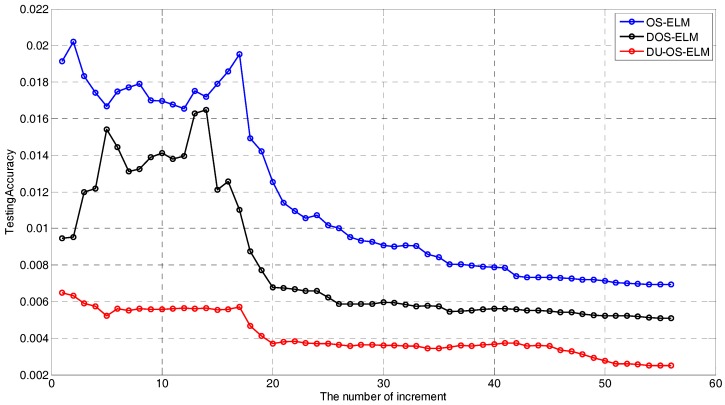
Change trends comparison among OS-ELM, DOS-ELM and DU-OS-ELM.

The convergence of DU-OS-ELM is analyzed theoretically in Section 3.3, and a simulation evaluation is also performed to show the convergence speed among DU-OS-ELM, DOS-ELM and OS-ELM. The number of initial training data $N_{0}$ has been taken as 100 and the size of block of data learned in each step is 20 in this simulation. Figure 8 details the changing trends among DU-OS-ELM, DOS-ELM and OS-ELM. According to this figure, as the number increment proceeds, the testing error tends to decrease, and DU-OS-ELM obtains the smallest testing error with the fastest convergence speed compared with DOS-ELM and OS-ELM, which illustrates that the proposed DU-OS-ELM has better dynamic tracking ability due to the utilization of the DFF and the USS. DOS-ELM tends to be stable when the number of increment approximately equals to 35 in the black line. However, OS-ELM tends to be stable when the number of increments is approximately equal to 50 in the blue line. The comparison results show that DOS-ELM has faster convergence speed than OS-ELM. In addition, we can find that the red line is smoother than others, which implies that DU-OS-ELM is more suitable for time-varying environments. According to the aforementioned analysis, the proposed DU-OS-ELM has faster convergence speed and is more effective for the time-varying environments.

**Figure 9:**
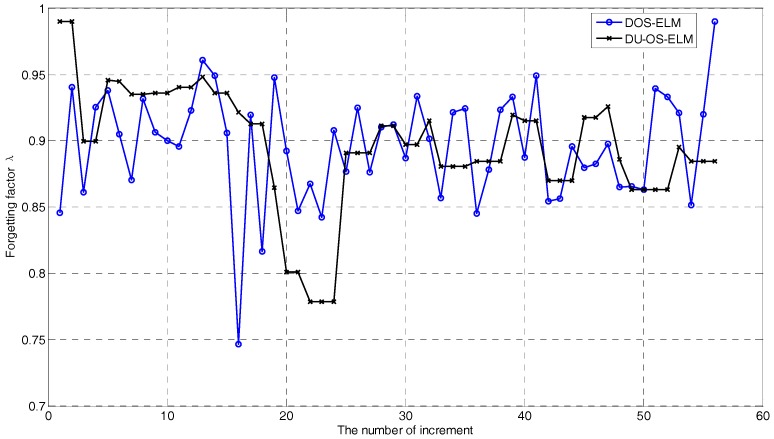
$\lambda_{k}$ by DOS-ELM and DU-OS-ELM.

The changes of the DFF $\lambda_{k}$ in Equation (19) of DU-OS-ELM (red line) and DOS-ELM (black line) are depicted in Figure 9. We can find that the DFF of DOS-ELM is updated iteratively depending on the predicted error. However, due to the addition of the USS, the DFF of DU-OS-ELM is not updated in each iteration, for example, there are constant segments when the number of increments equals 23, 34, 50, respectively. This proves the effectiveness of the USS and indicates that there is no need to update the model in each time step. In addition, according to the changes of the DFF, we can understand the changing status of the production process.

**Figure 10:**
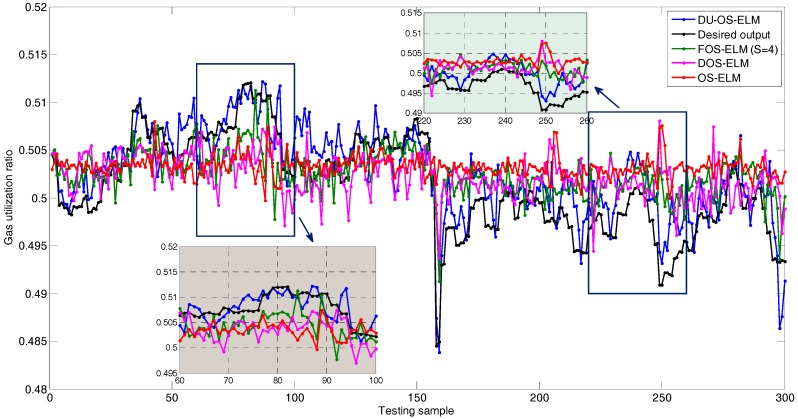
Simulation results of different approaches.

Figure 10 shows comparison results of DU-OS-ELM, DOS-ELM, FOS-ELM, and OS-ELM in a single trial. The parameter is set as *s* = 4 in FOS-ELM. The number of initial data is 100 and the size of block of data learned in each step is 10 in DU-OS-ELM, DOS-ELM and OS-ELM. The black line is the actual output value. As observed from Figure 10, the predicted results of the four approaches are basically consistent with the actual trend, which can reflect the changing trend of GUR, but there are differences in the agreement degree. Obviously, the predicted result of OS-ELM shows the lowest agreement with the actual values. The predicted results of DOS-ELM, FOS-ELM and OS-ELM have little difference, but DU-OS-ELM is better than the other three approaches. In order to identify the simulation results more clearly, the results between 60 and 100 and between 220 and 250 are magnified. As can be found from local amplified drawing in Figure 10, DU-OS-ELM is more close to the actual output value compared with other three approaches, which shows that the proposed DU-OS-ELM provides more accurately predicted results.

**Figure 11:**
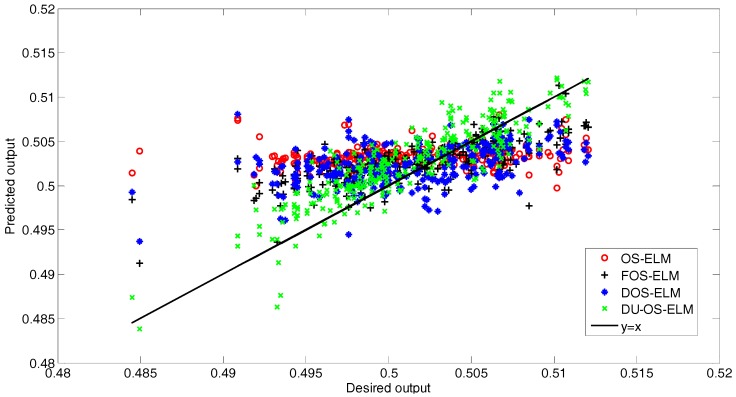
The correlation between the predicted values and desired values.

Figure 11 presents the correlation between the predicted values of the different approaches and the desired values. As observed from Figure 11, most of the predicted values of the proposed DU-OS-ELM are close to *y* = *x*, but the predicted values of the other approaches are relatively far away from *y* = *x*. In addition, the calculated results show that the correlation coefficient of DU-OS-ELM, DOS-ELM, FOS-ELM and OS-ELM equal to 0.8813, 0.7301, 0.7275 and 0.6613, respectively, which indicates that the proposed DU-OS-ELM has better prediction performance than others.

In order to evaluate the performance of the proposed DU-OS-ELM quantitatively, the following frequently criteria are used in the experimental results: training time, testing time, training RMSE, testing RMSE, mean absolute percentage error (MAPE) [48] and SD [6,7,15]. RMSE makes an excellent general purpose error metric for prediction model. MAPE is a relative value and usually expresses accuracy as a percentage. The smaller the RMSE and MAPE, the better the prediction accuracy is. SD is a measure of the dispersion degree of many experiment results. The smaller the SD, the more stable the algorithm is. The mathematical representations of the three statistics are shown in Equations (A4)–(A6) in Appendix C.

The performance of the proposed DU-OS-ELM is evaluated by one-by-one learning mode and chunk-by-chunk learning mode with 5 and 10 chunk sizes. Fifty trials are carried out in each case. The results are averaged and summarized in Table 4. According to Table 4, the training time decreases as the chunk size increases. In addition, it is worth noting that the testing accuracy only has a little change as the chunk size increases. For the same learning mode, the training time (learning time) of DU-OS-ELM is relatively longer than DOS-ELM due to the utilization of the USS, but the output weight is not always updated, thus, the training time of DU-OS-ELM does not increase obviously. Overall, the predicted accuracy of DU-OS-ELM is better than DOS-ELM. Meanwhile, both of the two approaches have smaller SD, however, DU-OS-ELM is smaller than DOS-ELM. It shows that the former is more stable than the latter. The above analysis once again proves the effectiveness of the USS in DU-OS-ELM for time-varying environments.

The compared results of different approaches are given in Table 5. Since batch ELM does not have the ability to learn sequentially, we use the entire training set to train, and other approaches are updated with the new data online. A fixed chunk size of 10 is selected for DU-OS-ELM, DOS-ELM and OS-ELM in the chunk-by-chunk learning mode. It can be found from Table 5 that DU-OS-ELM achieves the best generalization performance with the smallest RMSE and MAPE compared with other approaches. In terms of training time, batch ELM takes the least time. After adding DFF and USS, the training time of DU-OS-ELM is a little longer than that of FOS-ELM, but shorter than that of OS-ELM, which shows that DU-OS-ELM does not add too much computational complexity and is easy to implement. In addition, the time consumption of DU-OS-ELM is acceptable in actual production. Therefore, DU-OS-ELM can achieve the accurate GUR prediction, and can satisfy the production requirements.

## 6. Discussion

The motivations of the proposed DU-OS-ELM are mainly in the following three aspects:

(1) Give more attention on the newly acquired data and forget the old collected data gradually.
(2) Save storage space for matrix and avoid data saturation problem.
(3) Improve the dynamic tracking ability, reduce the time consumption and avoid the errors accumulation.

Therefore, the main contributions of the proposed DU-OS-ELM include: (1) a novel DFF with low computational complexity depending on the estimation errors is presented to improve the dynamic tracking ability in time-varying environments. The trained model can more closely track dynamic changes of data; (2) the USS is developed to judge whether the model should be updated with the newly coming data in each time step to avoid the errors accumulation and reduce the time consumption; (3) an online soft-sensor model is established based on the proposed DU-OS-ELM to implement the fast and accurate GUR prediction in BF ironmaking process. In order to further illustrate the superiority of the proposed approach, the characteristics including advantage and limitation of various approaches (including ELM, OS-ELM, FOS-ELM, DOS-ELM and DU-OS-ELM) are summarized in Table 6.

As observed from Table 6, the proposed DU-OS-ELM approach belongs to online learning with forgetting mechanism and update strategy for non-stationary or time-varying environments. However, according to the above experiments, the predicted results of DU-OS-ELM depend on the step size μ in Equation (19). A reasonable μ needs to be determined. It is worth noting that DU-OS-ELM becomes OS-ELM when λ=1. Thus, OS-ELM can be seen as a special case of DU-OS-ELM when all the observations are treated equally. In addition, a fixed sliding window, i.e., the collected old data are given 0 weight and the newly acquired data are given 1 weight in active area, is added in the FOS-ELM. From an extreme point of view, FOS-ELM can also be regarded as a special case of DU-OS-ELM.

The GUR soft-sensor model can reflect well the strong nonlinear relationships between the energy consumption and operating parameters, which is difficult to describe by any mechanistic model due to the high complexity of the reactions occurring inside a furnace. In addition, the application of this work will help BF operators judge the changes of energy consumption indicator in time properly, which provides a guide for them to determine the direction of controlling BF in advance.

## 7. Conclusions

This paper proposes an effective online learning approach named DU-OS-ELM with better generalization performance in the GUR soft-sensor model. In the proposed DU-OS-ELM, the novel DFF and USS are developed under the framework of OS-ELM to improve the performance in time-varying production environments. The DFF can automatically adjust with the dynamic change of production process to improve the dynamic tracking ability of the model. On the other hand, the USS is added to further meet the actual production situation. The real production data are employed to validate the effectiveness of the proposed DU-OS-ELM. The comparison experimental results indicate that the GUR soft-sensor model based on the proposed DU-OS-ELM has higher accuracy and smaller SD than others. The soft-sensor model can provide effective decision support for the optimization operation, energy savings and emission reduction of BF ironmaking processes.

## Figures and Tables

**Table 1:** Input variables description and correlation analysis results.

Variable Name	Unit	Correlation Grades
Blast volume	m^3^/min	0.8662
Blast temperature	°C	0.9237
Blast pressure	kPa	0.8795
Top pressure	kPa	0.7064
Top temperature	°C	0.8276
Permeability index	m^3^/min·kPa	0.7856
Oxygen enrichment	wt%	0.8136
Blast velocity	m/s	0.4844
Pulverized coal injection	t	0.5023
Thermal load	kJ/h	0.4087

**Table 2:** Statistical properties of GUR in terms of divided training and testing set.

Set	Maximum	Minimum	Mean	SD
Train	51.208	47.651	50.271	0.4404
Test	50.574	47.879	49.476	0.5379

**Table 3:** Statistical properties of the variables.

No.		Variable	Maximum	Minimum	Mean	SD
input	1	Blast temperature	1180.4	1144.4	1163.6	7.29
	2	Blast pressure	347.11	309.72	337.36	4.44
	3	Blast volume	4353.2	4199.4	4287.0	20.98
	4	Top pressure	190.40	177.83	185.38	1.74
	5	Top temperature	305.49	95.35	182.52	36.30
	6	Permeability index	0.81	0.58	0.75	0.0277
	7	Oxygen enrichment	44.79	37.68	41.24	1.1469
output	1	Gas utilization ratio	51.208	47.651	50.112	0.561

**Table 4:** Comparison results of DU-OS-ELM and DOS-ELM.

Algorithm	Learning Mode	Training Time (s)	RMSE		SD		MAPE	#nodes
			Training	Testing	Training	Testing		
DOS-ELM	1 by 1	0.1053	0.0031	0.0034	5.8480 × 10^−4^	7.6142 × 10^−4^	0.0622	20
	5 by 5	0.0406	0.0030	0.0033	5.4541 × 10^−4^	7.2880 × 10^−4^	0.0629	20
	10 by 10	0.0257	0.0032	0.0036	7.4076 × 10^−4^	6.4927 × 10^−4^	0.0637	20
DU-OS-ELM	1 by 1	0.1193	0.0029	0.0032	5.0033 × 10^−4^	6.5348 × 10^−4^	0.0611	20
	5 by 5	0.0616	0.0030	0.0034	4.9858 × 10^−4^	6.4049 × 10^−4^	0.0626	20
	10 by 10	0.0328	0.0029	0.0033	4.2275 × 10^−4^	5.0429 × 10^−4^	0.0619	20

**Table 5:** Comparison results of different approaches.

Algorithm	Training time(s)	RMSE		SD		MAPE	#nodes	Remark
		Training	Testing	Training	Testing			
ELM	0.0156	0.0068	0.0076	2.6834 × 10^−5^	7.3714 × 10^−5^	0.1086	20	batch
OS-ELM	0.0413	0.0055	0.0064	3.5139 × 10^−5^	9.3708 × 10^−5^	0.0822	20	10 by 10
FOS-ELM	0.0281	0.0043	0.0051	5.3216 × 10^−4^	7.7670 × 10^−4^	0.0794	20	s = 4
DOS-ELM	0.0257	0.0032	0.0036	7.4076 × 10^−4^	6.4927 × 10^−4^	0.0637	20	10 by 10
DU-OS-ELM	0.0328	0.0029	0.0033	4.2275 × 10^−4^	5.0429×10^−4^	0.0619	20	10 by 10

**Table 6:** Characteristics comparison of different approaches.

Algorithm	Learning Mode	Forgetting Mechanism	Update Strategy	Data Saturation	Application Scope	Limitation
ELM	Batch learning	-	-	-	Finite number of samples	The predicted results are limited by the initial training samples
OS-ELM	Online learning	No	No	Yes	Real-time and stationary situations	All the observations are treated equally
FOS-ELM	Online learning	Fixed sliding window	No	No	Short-term online prediction	The old data are discard directly when the new ones arrived
DOS-ELM	Online learning	Adaptive forgetting factor	No	No	Nonstationary or time-varying environments	Step size μ needs to be reasonably defined
DU-OS-ELM	Online learning	Adaptive forgetting factor	Yes	No	Nonstationary or time-varying environments	Step size μ needs to be reasonably defined

**Figure A1:**
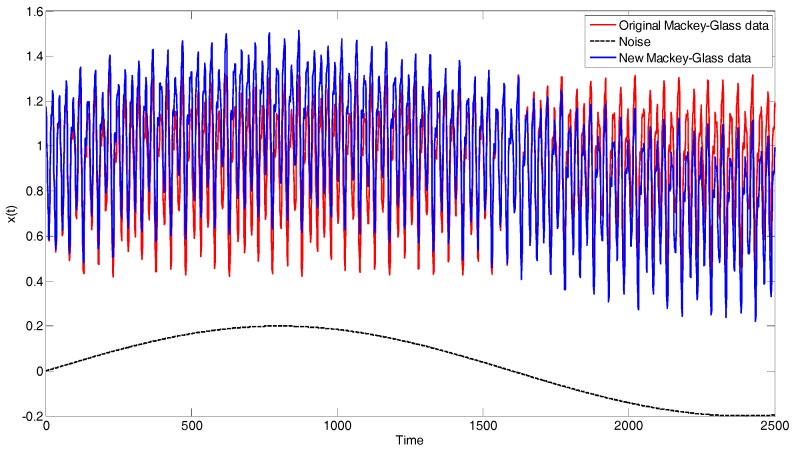
Mackey-Glass chaotic time series.

**Figure A2:**
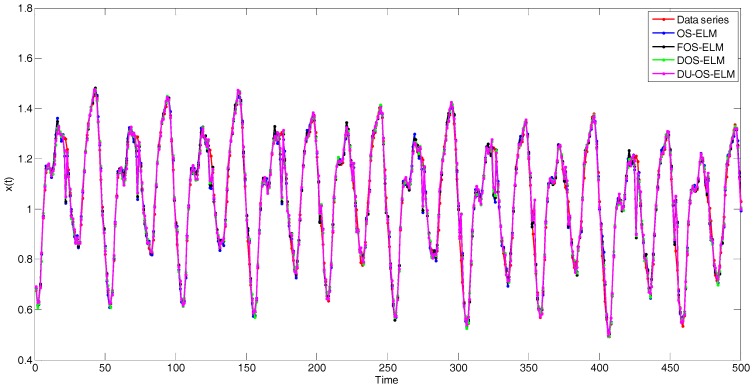
Simulation results of Mackey-Glass chaotic time series.

**Figure A3:**
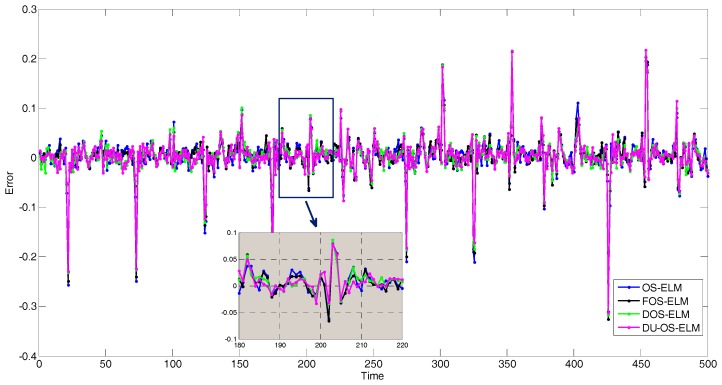
Error results of Mackey-Glass chaotic time series.

**Figure A4:**
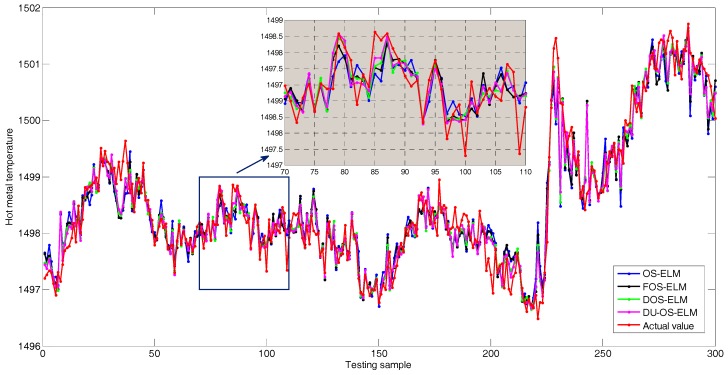
Prediction results of hot metal temperature based on different algorithms.

**Figure A5:**
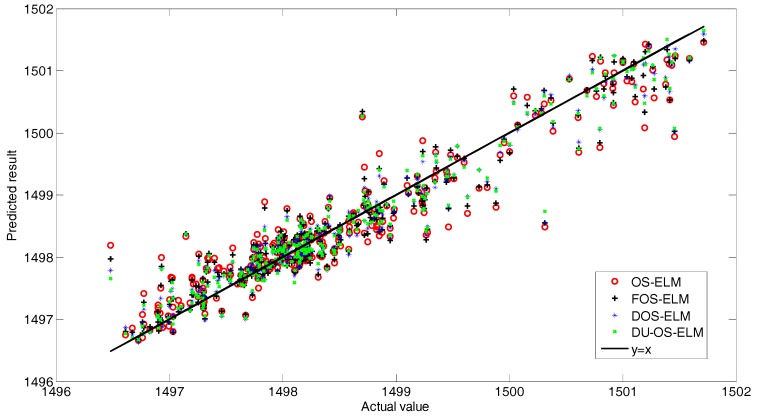
The correlation between the predicted values and actual values.

## Appendix A

In this appendix, we verify the effectiveness of the proposed DU-OS-ELM using the well-known Mackey-Glass chaotic time series. The data are generated by the following time-delay differential equation [15,20]:

(A1) $$\frac{dx(t)}{dt}=\frac{0.2x(t-\tau )}{1+x^{10}(t-\tau )}-0.1x(t),$$

where x(t) is the output value at time t, τ is delay time and τ=17. We set the initial value x(0)=1.2 and x(t)=0 for t<0. In order to create the time-vary time series, a sinusoid with amplitude 0.2 is added to the series as shown in Figure A1. We predict the output y(t)=x(t+6) from the past values of this time series, that is, x(t), x(t−6), x(t−12), x(t−18), so the data format is:

(A2) [x(t),x(t−6),x(t−12),x(t−18);x(t+6)].

We collect 2200 data pairs of the above format. The first 1700 input-output instances are used for training while the others are used for testing. In this simulation, OS-ELM [15] and FOS-ELM [21] are conducted for comparison. The number of initial data is 200 and the size of block of data learned in each step is 10 in DU-OS-ELM, DOS-ELM and OS-ELM. The parameter is set as *s* = 4 in FOS-ELM. Figure A2 shows the simulation results of Mackey-Glass Chaotic Time Series with different approaches. Figure A3 presents the error comparison results of different approaches. In order to identify the simulation results more clearly, the results between 180 and 220 are magnified. As can be found from local amplified drawing in Figure A3, DU-OS-ELM has less error compared with other three approaches, which shows that the proposed DU-OS-ELM presents more accurate predicted results on Mackey-Glass chaotic time series data.

The performance comparison results are shown in Table A1. As observed from Table A1, DU-OS-ELM achieves the best performance with the smallest RMSE and MAPE. The time consumption of DU-OS-ELM is similar to others. Therefore, the addition of DFF and USS does not bring computational burden.

**Table A1:** Comparison results of different approaches.

Algorithm	Training Time (s)	RMSE		MAPE	#Nodes	Remark
		Training	Testing			
ELM	0.5148	0.0461	0.0441	0.0296	200	batch
OS-ELM	1.2792	0.0445	0.0432	0.0274	200	10 by 10
FOS-ELM	1.2366	0.0436	0.0420	0.0229	200	*s* = 4
DOS-ELM	1.2324	0.0420	0.0404	0.0198	200	10 by 10
DU-OS-ELM	1.2480	0.0411	0.0398	0.0183	200	10 by 10

## Appendix B

Another important issue in the BF ironmaking process is the hot metal temperature prediction. A soft-sensor model of hot metal temperature is established to further prove the contribution of the proposed DU-OS-ELM in this appendix. Experiments are reported to demonstrate the performance and effectiveness of the proposed approach.

Hot metal temperature is used as a chief indictor of the thermal status inside the BF, which determines the quality of the resulting hot metal. Therefore, it needs to be controlled within a certain range. However, the measurement of hot metal temperature can be implemented only off-line by soft-sensor methods, so the result is lagging behind and against the real-time performance of hot metal temperature monitoring [49,50]. The effective soft-senor approach is a good choice to establish the hot metal temperature prediction based on the easily measured parameters. Additionally, BF ironmaking is a typical dynamic and time-varying production process with hundreds of physical and chemical reactions occurring [37]. The prediction model based on fixed learning samples cannot satisfy the needs of online use. Besides, all the data treated equally may not be sufficient to track all the system dynamics. For actual production situation, when the system is relatively stable or the current training model can meet the prediction requirement, it may not need to update in each time step. Therefore, the proposed DU-OS-ELM is more suitable for establishing the prediction model of hot metal temperature.

Six relevant variables, including blast volume, blast temperature, blast present, pulverized coal injection, oxygen enrichment and permeability index, are selected as input variables. The hot metal temperature is the only output variable. We also collect 1500 data pairs in all. The first 1200 input-output instances are used for training while others are used for testing to evaluate the performance of the model. In order to make comparison, we also use the OS-ELM, FOS-ELM, DOS-ELM and DU-OS-ELM to build the prediction model of hot metal temperature. The simulation results of these learning approaches are shown in Figure A4. In order to identify easily, we magnify the results between 70 and 110, where one can found that DU-OS-ELM can track the actual value better than other three approaches. Simultaneously, the correlation between the predicted values of the different approaches and actual values are demonstrated in Figure A5 to further illustrate the superiority of the proposed approach. As observed from Figure A5, the predicted values of the proposed DU-OS-ELM is closer to y = x than those of other approaches. The correlation coefficient of DU-OS-ELM, DOS-ELM, FOS-ELM and OS-ELM equal to 0.9621, 0.9579, 0.9087 and 0.8751, respectively, which indicates that DU-OS-ELM has better prediction performance than the other methods. Table A2 summarizes the performance comparison results. From Table A2, the results also show that DU-OS-ELM achieves the best performance. According to the above experimental results, DU-OS-ELM can achieve more accurate online prediction of hot metal temperature. Thus, compared with other approaches, DU-OS-ELM is more suitable for hot metal temperature in time-varying environments. In addition, the results can guide the BF operators to take reasonable measures (including control span and the control direction) in the next step.

**Table A2:** Comparison results of different approaches.

Algorithm	Training Time (s)	RMSE		MAPE	#nodes	Remark
		Training	Testing			
ELM	1.1700	0.0685	0.0754	0.1442	500	batch
OS-ELM	4.6176	0.0621	0.0688	0.1284	500	10 by 10
FOS-ELM	4.3992	0.0603	0.0638	0.1166	500	*s* = 4
DOS-ELM	4.2900	0.0536	0.0561	0.0961	500	10 by 10
DU-OS-ELM	4.3212	0.0527	0.0548	0.0922	500	10 by 10

## Appendix C

List of related equations:

Data normalization processing method:

(A3) $$x_{k}=\frac{{\tilde{x}}_{k}-\min({\tilde{x}}_{k})}{\max({\tilde{x}}_{k})-\min({\tilde{x}}_{k})},$$

where ${\tilde{x}}_{k}$ and $x_{k}$ represent the *k*th variables values before and after change respectively, $\max({\tilde{x}}_{k})$ and $\min({\tilde{x}}_{k})$ are the maximum and minimum values of the *k*th variable before normalized.

RMSE:

(A4) $$RMSE=\frac{1}{50}\sum_{k=1}^{50}\left(\sqrt{\frac{\sum_{i=1}^{n}{\left(y_{i}-{\hat{y}}_{i}\right)}^{2}}{n}}\right),$$

MAPE:

(A5) $$MAPE=\frac{1}{n}\sum_{i=1}^{n}\frac{\left|y_{i}-{\hat{y}}_{i}\right|}{y_{i}}\times 100\%,$$

SD:

(A6) $$SD=\sqrt{\sum_{i=1}^{n}\frac{{\left(\left|\frac{y_{i}-{\hat{y}}_{i}}{y_{i}}\right|-\bar{e}\right)}^{2}}{n}},$$

where *k* is the *k*th trial, *n* is the number of the data in the testing set, $y_{i}$ is the actual value, ${\hat{y}}_{i}$ is the predicted value, and $\bar{e}$ is the average value of the relative error.
